# Osteoplastic Amputations

**Published:** 1904-10-08

**Authors:** 


### OSTEOPLASTIC AMPUTATIONS.

In the days before anæsthetics and antiseptics were used, a large part of the surgeon's operative experience was confined to amputations of the extremities, and this branch of work was perhaps brought as nearly to the height of perfection as was possible with the limitations imposed by the necessity for rapidity of procedure and the certain expectation of ensuing sepsis. Now, however, the surgeon is not only able to carry out carefully-planned and prolonged operations, but in most cases he may rely upon aseptic healing of the wound. With these two new principles to help, it would not have been surprising to observe a corresponding alteration of technique; and to some extent this has occurred; for example, transfixion methods and thick muscular flaps have given place to skin flaps with circular division of muscle. So far some improvement must be admitted; but it is none the less true that there still remains much room for advancement in the technique of amputations, a fact to which the large proportion of tender and painful stumps seen in out-patient rooms and elsewhere bears eloquent testimony. No doubt the superior fascinations of abdominal surgery and the great reduction of cases in which amputation is now necessary, thanks to the assistance rendered to conservative treatment by antiseptic methods, may be adduced to explain the anomaly. Explanations and excuses, however, can be placed on one side, and while urging that a more critical study should be given to the general subject of amputations, and more care should be devoted to following up such cases after they leave hospital, attention may usefully be drawn to the conclusions of Bier. The latter was able to convince himself that the surest way to produce a stump free from pain and tenderness, and which would endure prolonged pressure was to cover the sawn surface of the divided bone with an osteoplastic flap, or, if this were impossible, to perform a disarticulation. It has been recognised widely enough that the amputations associated with the names of Pirogoff, Stephen Smith, and Stokes and Gritti leave stumps which are eminently satisfactory, but the beneficial results have been more commonly attributed to the nature of the flaps and other factors than to the main one enunciated by Bier. Moschcowitz[1] points out that failure to produce good functional stumps has been attributed from time to time and by various writers to most of the structures comprising the stump, viz. : (1) The integument, (2) the muscles, tendons, and fasciæ ; (3) the nerves, (4) the cicatrix, (5) the bone and periosteum. He further shows that whether the skin in question be naturally adapted to withstand pressure or not it will very soon become so on being put to that use. Both the nerves and the scar tissue may be in part or wholly responsible for failure ; but here prophylaxis is easy, for the nerves should be isolated during the operation, drawn out as far as possible, and divided at the upper limit, and the skin incision arranged, if possible, so that the cicatrix will not lie exactly over the end of the divided bone ; but he does not place very great stress on the latter point, and does not think it is ever of sufficient importance to lead the sugeon to sacrifice any of the length of the stump to it, because the consideration has application almost solely to cases which do not heal aseptically. Practical experience has led him to rely upon the absolute correctness of Bier's dictum quoted above, which he believes to be the basis of one of the greatest advances of modern surgery and to be worthy of more general consideration than it has up till now received.

[1] Med. News, August 27.

